# A modified surgical approach to induce circle Willis perforation in mice using the common carotid artery

**DOI:** 10.1038/s41598-025-97603-1

**Published:** 2025-04-21

**Authors:** Rui Zhang, Dilaware Khan, Sajjad Muhammad

**Affiliations:** 1 Department of Neurosurgery, Medical Faculty, University Hospital Düsseldorf, Heinrich- Heine-Universität, Mooren Str. 5, 40225 Düsseldorf, Germany; 2 Department of Neurosurgery, University of Helsinki, Helsinki University Hospital, Helsinki, Finland; 3 Department of Neurology, First Medical Center of PLA General Hospital, PLA Medical College, Beijing, China

**Keywords:** Common carotid artery approach, Circle Willis perforation, Needle puncture, Subarachnoid hemorrhage mouse model, Translational stroke research., Stroke, Cerebrovascular disorders, Experimental models of disease

## Abstract

The Circle of Willis perforation (cWp) mouse model is widely used in subarachnoid hemorrhage (SAH) research but involves sacrificing the external carotid artery (ECA), which may have a potential affect on the hemodynamic in the carotid arteries and cortical perfusion. We propose a modified approach using needle puncture via the common carotid artery (CCA) to preserve carotid vascular integrity. Twenty-seven C57BL/6 mice were randomly assigned into three groups and underwent cWp surgery in three procedures: sham (*n* = 3), ECA (*n* = 12), and CCA (*n* = 12). Surgical duration, success rate, intraoperative intracranial pressure (ICP) fluctuations, 24-hour mortality, and neurological deficits were assessed. The CCA approach achieved a 100% success rate and shorter surgical duration than the ECA approach (ECA 73 ± 18 vs. CCA 36 ± 10 min, *P* < 0.05). ICP fluctuations and mortality rates were comparable between the ECA and CCA groups (*P* > 0.05), indicating that the CCA approach shared a similar pattern with the ECA approach. Neurological outcomes were similar across SAH groups (CCA SAH induction and ECA SAH induction) but worse than sham (*P* < 0.05) in terms of body weight loss, open-filed test and Rotarod test performances. This modified cWp CCA approach, which preserves the carotid structures, helps eliminate hemodynamic bias and offers a potentially more efficient alternative with a shorter surgical duration compared to the classical ECA approach. It may prove to be a valuable option for broader application in SAH preclinical research.

**Supplementary Information:**

The online version contains supplementary material available at 10.1038/s41598-025-97603-1.

## Introduction

Spontaneous subarachnoid hemorrhage (SAH) represents a devastating pathology within the central nervous system with very high mortality and morbidity killing around 30–50% of affected patients^1–3^. In 2021, the incidence of SAH was found to be 37.09% higher than that in 1990, and the age standardized incidence rates showed a decreased trend^4^. Despite its clinical significance, our understanding of the pathophysiological changes that ensue after the onset of this condition remains limited^5^.

At the heart of this study lies the SAH animal model, a crucial tool for simulating the disease, investigating its mechanisms, and developing effective interventions. In recent years, two prominent SAH modeling approaches have emerged: the injection model and the endovascular perforation model. The circle Willis perforation (cWp) model has gained favor for closely mimicking the natural process of human aneurysm rupture compared to the injection model^6,7^.

However, the classical cWp model through the ECA approach has drawbacks, requiring the sacrifice of the ECA, leading to a deficit of the vascular structure^8,9^. The sacrifice of the ECA results in the diversion of blood flow towards the ICA, thereby altering hemodynamic within the ICA. This phenomenon has been demonstrated in models of intracranial aneurysms by Aoki et al.^10^and more recently by our research group^11^. Although ECA sacrifice is generally well-tolerated in mice, the resultant hemodynamic changes may introduce variables that could confound studies investigating the pathophysiology of SAH. Additionally, the ECA route presents a more complex anatomical course than the CCA, potentially offering a steeper learning curve for novice surgeons. To address these issues, we propose a modified surgical approach via the CCA to induce SAH in the cWp mouse model including puncture on the CCA, and absence of sacrificing the ECA, thus reducing the manipulation complex and preserving all the normal vascular structures.

## Results

### Intraoperative intracranial pressure monitoring, and surgical duration

**Fig. 1:**
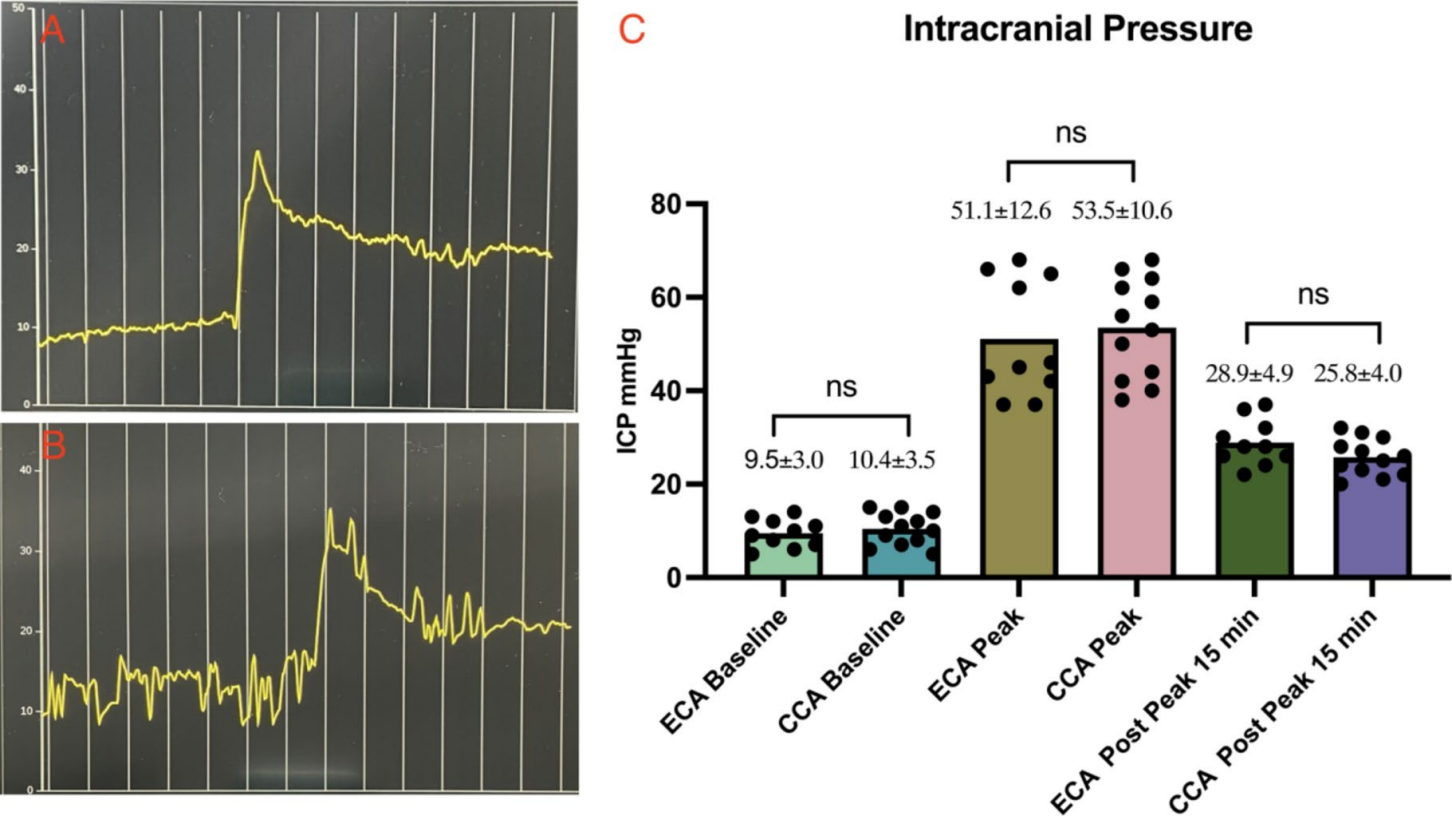
Intraoperative Intracranial Pressure Monitoring and Data Comparison. **A** illustrates the intraoperative ICP fluctuation recording in the ECA group, and **B** depicts a similar fluctuation for the CCA group. Both groups exhibited the characteristic curve of ICP elevation after induction of SAH. **C**: One-way ANOVA analysis revealed no significant differences in ICP baseline values, peak values, and post-peak 15-minute values in the ECA and CCA groups.

**Fig. 2:**
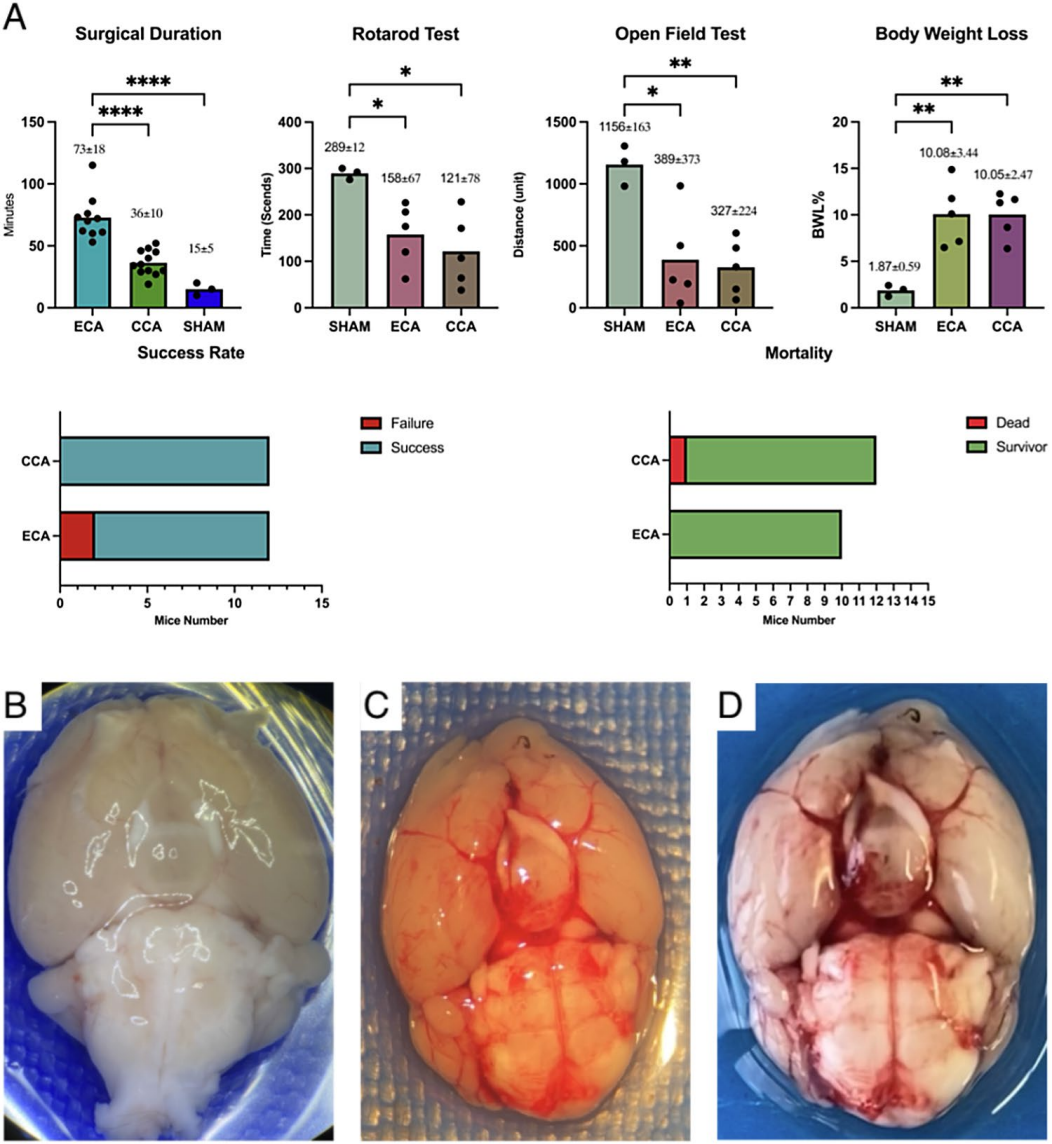
Surgical Duration, Mortality, Success Rate, Postoperative Neurological Assessment, and Autopsy Findings. **A**: Surgical duration is significantly shorter in CCA than in ECA (*P* < 0.05). Postoperative assessments show deterioration performance in ECA and CCA groups compared to Sham (*P* < 0.05), with no significant differences between the ECA and CCA groups. Two ECA mice had filament insertion failure; One mouse from CCA group died on postoperative day one. The success rate and mortality in those two groups without significant difference. Autopsy result: Sham(Fig. 2B), ECA SAH induction(Fig. 2C), CCA SAH induction(Fig. 2D).

In both the ECA and CCA groups, a noticeable cliffy elevation in ICP has been observed post-successful perforation. The baseline of ICP in ECA and CCA groups was 9.5 ± 3.0 vs. 10.4 ± 3.5 mmHg, with a *P* > 0.05. Peak ICP values were comparable between the two groups (ECA 51.1 ± 12.6 vs. CCA 53.5 ± 10.6 mmHg, *P* > 0.05), and 15 min after the peak, ICP dropped to 28.9 ± 4.9 mmHg in the ECA group and 25.8 ± 4.0 mmHg in the CCA group, with a *P* > 0.05. The ICP monitoring illustrated a similar pattern in both the ECA and CCA groups. Surgical duration for ECA decreased from 115 min to 53 min with accumulating experience, while the CCA group exhibited a reduction from 52 min to 19 min. Data analysis revealed a significant difference between the two groups (ECA 73 ± 18 vs. CCA 36 ± 10 min, *P* < 0.05) (Table 1; Figs. 1 and 2).

**Table 1:** Summary of Results - Intraoperative intracranial pressure (ICP) monitoring, surgical duration, postoperative mortality, success rate, and neurological assessment.

Group	ICP Base (mmHg)	ICP Peak (mmHg)	ICP Post peak (mmHg)	Surgical Duration (Minutes)	Mortality (%)	Success Rate (%)	Rotarod Test (Seconds)	Open-field Test^‡^	Body Weight Loss (%)
CCA^Σ^	10.4 ± 3.5 *n* = 12	53.5 ± 10.6 *n* = 12	25.8 ± 4.0 *n* = 12	36 ± 10 *n* = 12	8.33	100	121 ± 78 *n* = 5	327 ± 224 *n* = 5	10.05 ± 2.47 *n* = 5
ECA	9.5 ± 3.0 *n* = 10	51.1 ± 12.6 *n* = 10	28.9 ± 4.9 *n* = 10	73 ± 18 *n* = 10	0	83.33	158 ± 67 *n* = 5	389 ± 373 *n* = 5	10.08 ± 3.44 *n* = 5
SHAM	--	--	--	15 ± 5 *n* = 3	0	--	289 ± 12 *n* = 3	1156 ± 163 *n* = 3	1.87 ± 0.59 *n* = 3

^‡^The results of open-field test were recorded by using absolute value of the moving distance without units.

^Σ^One mouse from the CCA group died on the postoperative day one, its intraoperative data was still valid.

### The surgical procedure success rate and post 24-hour mortality

In the Sham group, all three mice successfully underwent the procedure and survived post-surgery. However, in the ECA group, two mice experienced filament insertion failure into the ICA, resulting in a success rate of 83.33% (10 out of 12 mice). Conversely, all 12 mice in the CCA group successfully underwent SAH induction. Regarding 24-hour mortality, the Sham group had zero deaths out of three mice (mortality: 0%). In the CCA group, one out of 12 mice died, resulting in a mortality rate of 8.33%. While in the ECA group, there were no deaths among the 10 mice (mortality: 0%). No significant differences were observed between the ECA and CCA groups in terms of success rate and mortality, with a *P* > 0.05 (Table 1; Fig. 2).

### Postoperative neurological assessments and autopsy

We tested all three mice in the Sham group and randomly each five mice from CCA and ECA groups by Rotarod test, open-field test, and body weight loss on postoperative day one and compared them in groups. The body weight loss results from groups: Sham = 1.87 ± 0.59%; ECA = 10.08 ± 3.44%; CCA = 10.05 ± 2.47%. The Rotarod test results from groups: Sham = 289 ± 12 s; ECA = 158 ± 67 s; CCA = 122 ± 78 s. The open-field test results from groups: Sham = 1156 ± 163; ECA = 389 ± 373; CCA = 327 ± 224. Those post-SAH neurological assessment results were analyzed and compared via One-way ANOVA, indicating a deterioration in performance in ECA and CCA groups compared to Sham (*P* < 0.05), with no significant differences between the ECA and CCA groups. The ventral surface of the brain samples was checked, and both the ECA and CCA groups succeeded in SAH introduction (Table 1; Fig. 2).

## Discussion

In the realm of SAH research, animal models have proven to be indispensable tools for shedding light on the intricate mechanisms underlying the disease. While various species, including cats and dogs, have been employed in such studies, the murine model, encompassing both rats and mice, stands out as the most extensively utilized^12,13^.

Among the diverse methodologies available for inducing SAH in murine, including direct injection of blood into the cisterna magna^14^ or into the suprachiasmatic cistern^15^, tearing an intracisternal vein^16^, or perforating the Circle of Willis using an endovascular filament inserted through the ECA^8,9,17^.

The cWp model represents a modification derived from the Middle Cerebral Artery Occlusion (MCAO) model^18^. This model’s popularity can be attributed to its successful replication of human SAH onset conditions by the rupture of an artery within the Circle of Willis, this model effectively reproduces the dynamics of SAH pathogenesis, facilitating investigations into the associated biomarkers, sterile inflammations, neurological deficits, post-hemorrhagic outcomes, and exploration of therapeutic interventions^19^. While a modified version of the cWp model have been proposed^20^, the classical cWp model still retains the ECA as its primary insertion site. It’s remarkable to note that in this approach, the ECA must be sacrificed during the procedure, which may affect the hemodynamic changes, cerebral perfusion and shear stress in vascular wall impacting the post SAH pathophysiological mechanisms^21,22^.

Simultaneously, it’s worth noting that in the classical cWp model, where the ECA is chosen as an insertion site, the ECA itself needs to be dissected along the artery in its distal direction to create as much space as possible. This step is crucial for facilitating subsequent manipulations and ensuring the accuracy and effectiveness of the procedure. In some cases, the authors have also opted to sacrifice OA and PPA to create a sufficient surgical field for operation^9^. These complex manipulations underscore the demand for experienced microsurgical skills, as executing such intricate steps can present a challenging cliffy learning curve for researchers leading to a huge impact on the reproducibility of the experiment.

Simplifying the SAH modeling procedure is of paramount importance to enhance reproducibility and reduce confounders in translational SAH research. In this study, we introduced a modified surgical procedure for inducing SAH by directly puncturing the CCA and subsequently inserting the filament building upon the Seldinger technique^23^. In our study, an experienced neurosurgeon performed cWp surgeries using both the ECA and CCA approaches. A notable finding was the significantly reduced surgical duration in the CCA group, highlighting the efficiency of this modified method (ECA: 73 ± 18 min vs. CCA: 36 ± 10 min, *P* < 0.05). This can be easily understood, as the CCA approach eliminates the need for dissecting the complex vascular structure, sparing the OA, PPA, and ECA from separation and sacrifice, resulting in considerable time savings. The shorter surgical duration could reflect a more straightforward technique, potentially reducing the overall stress on the animal and minimizing anesthesia time.

The mortality of SAH mice was 4.5% on the post-SAH day one (1 of 22), aligning with findings in a previous report^7^. Regarding the success rate, two mice in the ECA group experienced difficulty inserting the filament from the ECA to the ICA. The anatomical configuration of the CCA, ECA, and ICA forms a “Y” shape, making filament insertion from the ECA to the ICA challenging due to the bifurcation. Although rotating the ECA stump, as suggested in prior literature, can help, overcoming this obstacle remains problematic^9^. However, with the CCA approach, inserting the filament along the CCA to the ICA provides a straight and smooth pathway, simplifying the procedure. Another potential challenge is the bifurcation between the PPA and ICA (Fig. S2). While previous study recommended sacrificing the OA and PPA for easier filament insertion^9^, we argue that such sacrifices are unnecessary. Despite the filament being occasionally misled towards the PPA due to the natural arterial angles, this error can be discerned by assessing the length of the inserted filament and monitoring in ICP reactions. With a 50% chance of success in each attempt, multiple trials usually lead to proper filament insertion without the need to dissect and sacrifice the OA and PPA. In theoretical terms, the ECA approach presents two obstacles—namely, the ECA-ICA bifurcation and PPA-ICA bifurcation—while the CCA approach involves only overcoming the PPA-ICA bifurcation. Although the success rates in both the ECA and CCA groups show no significant difference, the markedly increased complexity of the ECA approach significantly prolongs the surgical duration, as discussed earlier.

The postoperative neurological assessment demonstrated that both the ECA and CCA approaches effectively induced SAH in the mouse model, yielding comparable outcomes. In both the Rotarod and open-field test, SAH mice exhibited diminished movement and reduced balance abilities, additionally, body weight loss in SAH mice was significantly higher than that in sham groups, regardless of the induction method employed. These results are straightforward, emphasizing that the primary goal is to perforate the circle of Willis, and the insertion site is not the decisive factor. In achieving the essential target of inducing subarachnoid hemorrhage, both the ECA and CCA approaches yield comparable outcomes (Table 1; Fig. 2).

The CCA approach offers two eminent advantages in the context of SAH modeling. Firstly, it simplifies surgical manipulation, leading to significantly shorter surgical duration. Secondly, it preserves the integrity of all anatomical structures, eliminating the need for complex artery dissections. Crucially, our research has demonstrated that the CCA approach can generate a SAH model of the same quality as the ECA approach while reducing confounders and time of surgical procedure as well as the time for anesthesia. This underscores the potential of the CCA approach to serve as a streamlined and equally reliable alternative for cWp mouse model production, reducing surgical complexity while maintaining research rigor. However, the CCA approach does have a limitation to consider. One of the key reasons the ECA is preferred in murine models is that it can be easily sacrificed, and in case of complications, the opposite ECA can serve as an alternative. In contrast, if an issue arises during a procedure using the CCA, this artery cannot be sacrificed, which makes the ECA approach more flexible in certain situations.

In conclusion, this modified cWp CCA approach, which preserves the carotid structures, helps eliminate hemodynamic bias and offers a potentially more efficient alternative with a shorter surgical duration compared to the classical ECA approach. It may prove to be a valuable option for broader application in SAH preclinical research.

There are some limitations of this study, it primarily focuses on the surgical technique and its success in establishing a cWp mouse model. As an initial study, it serves to demonstrate the feasibility of the surgery itself. However, this work is only the beginning, and numerous questions remain unanswered. Further experiments are necessary to gain a deeper understanding of this modified surgical approach, particularly in comparison to the classical ECA method. Continued research will be essential to refine the technique and elucidate its implications for SAH pathophysiology.

## Materials and methods

### Ethical approval and experiment workflow

This study was conducted in accordance with the ethical standards outlined in the Animal Protection Act (§ 8 Abs. 1, TierSchG) and the Animal Welfare Experimental Animal Regulations (§ 31, TierSchVersV) of Germany. Approval for the experiment was granted by the Animal Care Committee of the District Government of North Rhine-Westphalia (Protocol Number: 81 - 02.04.2021.A195). All surgical procedures were performed under isoflurane anesthesia, with rigorous efforts to minimize animal suffering. Furthermore, the study adhered to the ARRIVE guidelines for animal research.

**Fig. 3:**
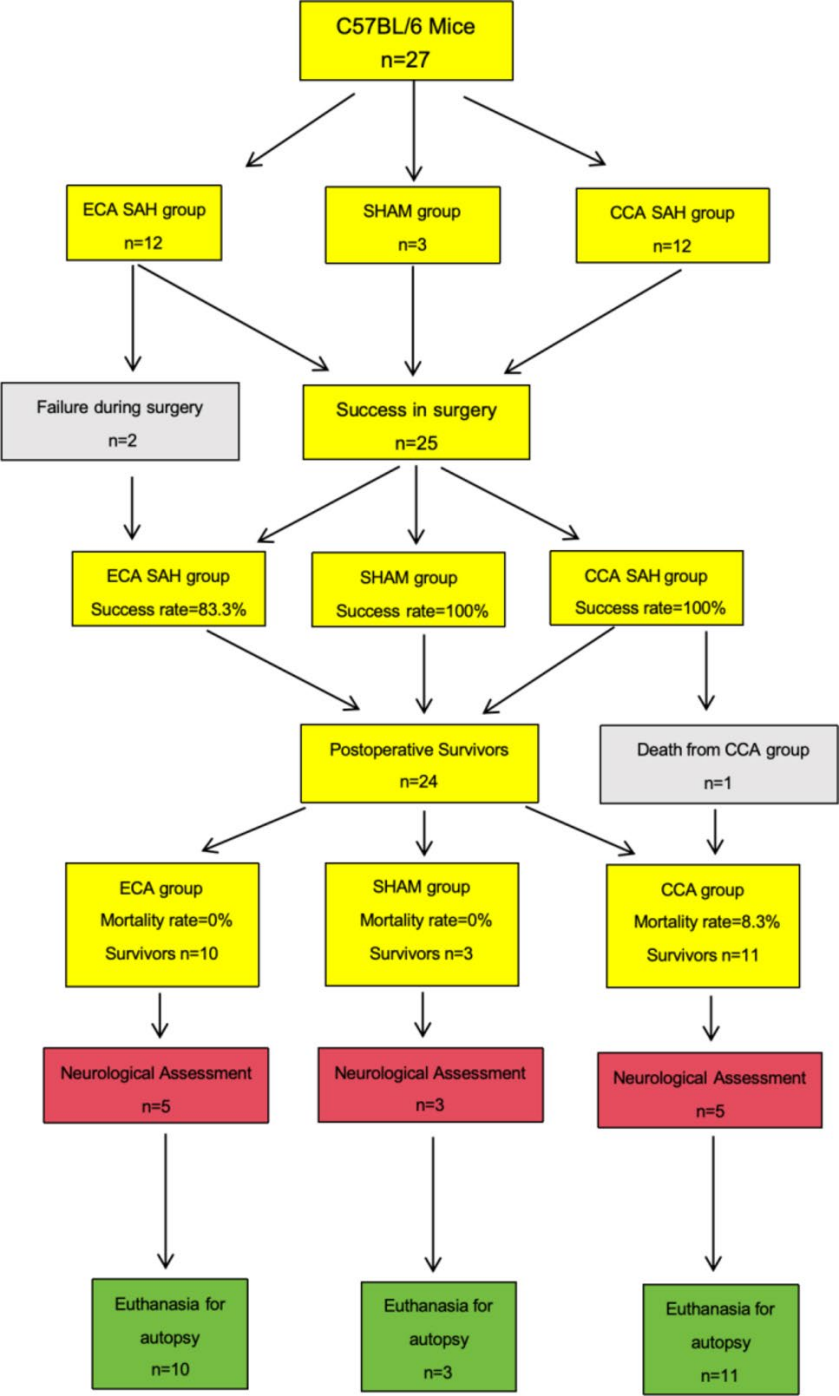
Workflow of “A Modified Surgical Approach with Preserved Carotid Artery to Induce Circle Willis Perforation Mouse Model”. Experimental plan comparing currently existing model (ECA group) to compare newly established model (CCA group). The cohort was subdivided into SHAM (n=3), ECA group (n=12), and CCA group (n=12). Two mice in the ECA group were excluded due to filament insertion failure. One mouse died from CCA group on the postoperative day one.

A cohort of 27 male *C57BL/6*wild-type mice, aged 4 months and weighing between 25.2 and 34.9 g, was utilized (Janvier Laboratory Le Genest-Saint-Isle, France). Surgical procedures were conducted to assess the feasibility of the modified CCA approach, with a comparative analysis against the conventional ECA approach. A proficient neurosurgeon conducted surgeries via the CCA approach. Additionally, the ECA approach was also performed by the same surgeon following the protocol described in the prior studies^8,9^. Another group of mice underwent the anesthesia and surgical procedure but without filament perforation served as SHAM group. Then the study cohort was subsequently categorized into three groups based on the type of surgery: SHAM (*n* = 3), ECA (*n* = 12), and CCA (*n* = 12), and the mice were randomly assigned into those groups preoperatively (Fig. 3).

### Perioperative management

Mice were fed in the human-controlled day/night rhythm, and free to food and water, before and after surgery. Standard microsurgical instruments, including 33 Gauge insulin injection needles and microsurgical forceps, were employed for the procedures (Fig. S1). The surgical microscope used provided a magnification ranging from 7 to 45 times (Leica). Anesthesia was administered to the mice, induced at 5% isoflurane and maintained at 1.5 ~ 2% isoflurane, delivered through a nose cone with 2 L Oxygen per minute. To maintain a constant body temperature of 37.5 °C, a feedback-controlled heating pad was utilized with a setting of 37.7 °C.

### Intraoperative intracranial pressure monitoring, and surgical duration

Intracranial pressure was monitored using a micro-pressure transducer from Raumedic, Germany (RAUMED Neurosmart). The sensor was positioned on the left parietal epidural space while the mice were prone. The surgical duration was recorded from the neck incision to the closure of this incision, excluding the ICP sensor implantation and post-SAH 15-minute observation.

### Surgical procedure

#### SHAM group surgical procedure

The Sham animals underwent anesthesia, during which the vascular structures including the CCA, ECA, ICA, and ECA-ICA bifurcation were exposed. However, no filament insertion was performed in the sham animals.

#### ECA approach surgical procedure

Briefly introduce the ECA approach as described previously: the animals were anesthetized and positioned in a supine orientation after the ICP monitor sensor implant. A midline incision was made to access the neck, and the left CCA was exposed, further dissection was made to expose ECA, ICA, occipital artery (OA), and pterygopalatine artery (PPA). Then tracked the ECA to its distal end as long as possible, ligated and cut the ECA, a 5 − 0 mono-filament (Prolene Ethicon) was carefully inserted through the ECA and guided into the ICA until it reached the vicinity of the anterior cerebral artery (ACA) and middle cerebral artery (MCA) bifurcation. The filament was then further advanced until a discernible and abrupt increase in ICP signified the successful induction of SAH (Fig. 1). Subsequently, the suture was retracted into the ECA, enabling complete perfusion of the ICA (Fig. S2). In our case, the PPA was not sacrificed as the previous paper described^8,9^.

#### CCA approach surgical procedure

**Fig. 4:**
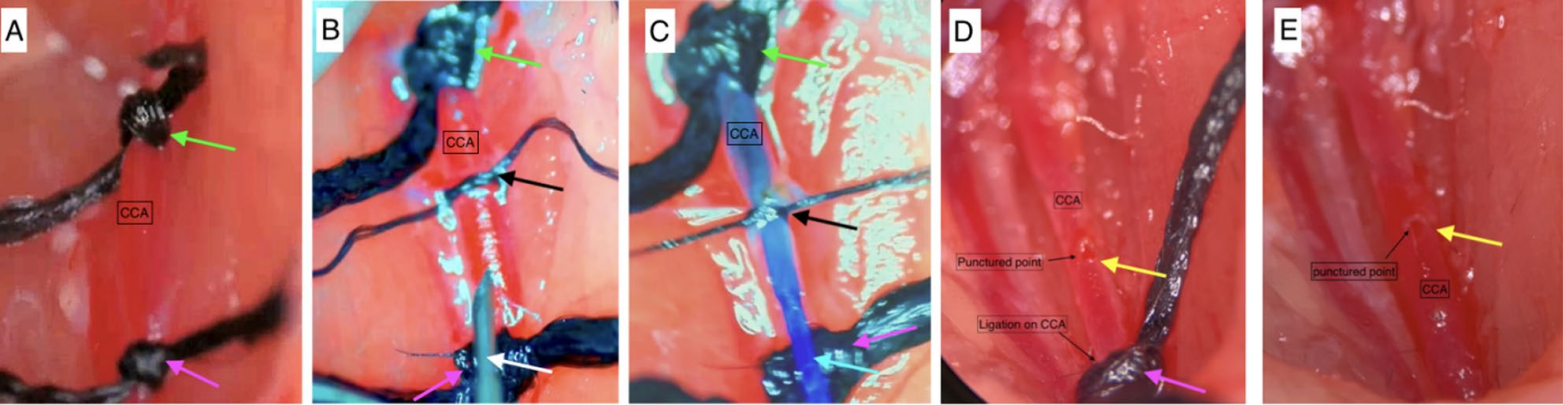
Sequential Steps of the Common Carotid Artery Approach for the Circle of Willis Perforation Subarachnoid Hemorrhage Model. **A**: Cervical segment of Common Carotid Artery (CCA) isolated, with proximal and distal ligation. **B**: 33-gauge needle punctures CCA; 5-0 Nylon filament inserted and fixed with middle ligation; distal ligation removed. **C**: 5-0 Nylon mono-filament inserted and fixed with middle ligation; distal ligation then completely removed. **D**: Middle ligation removed for blood perfusion via Willis circle; gradual release of proximal ligation to minimize rebleeding risk. **E**: Proximal ligation completely removed for full CCA lumen perfusion. Arrows: Pink-proximal ligation; Green-distal ligation; Black-middle ligation; White-needle; Blue-filament; Yellow-puncture site.

**Fig. 5:**
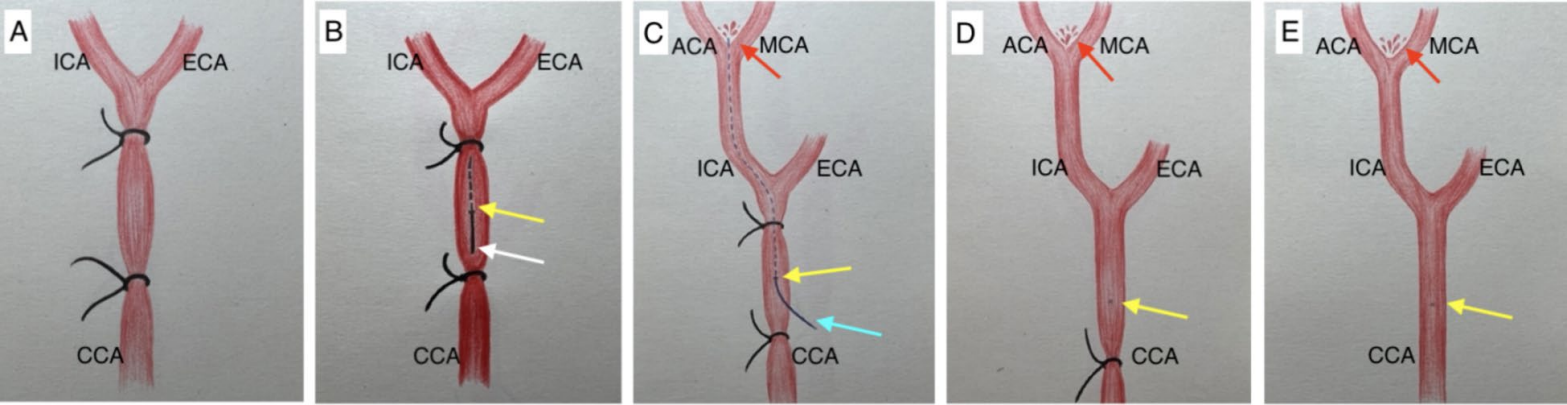
Schematic Sequential Steps of the Common Carotid Artery Approach for the Circle of Willis Perforation Subarachnoid Hemorrhage Model. Arrows: Yellow: Puncture site. White: 33 gauge needle; Sky blue: 5-0 Nylon mono-filament; Red: Anterior Cerebral Artery (ACA) and Middle Cerebral Artery (MCA) bifurcation perforation site.

The exposure of the left CCA was similar as described in ECA approach. However, there was no tracking and dissection of the ECA. After a meticulous dissection of the surrounding tissue and membranes enveloping the CCA, two ligations using 5 − 0 silk sutures (Ethicon) were performed. The first ligation was distal, placed at the bifurcation of the ICA and ECA, while the second ligation was proximal and positioned as close as feasible to the direction of the aortic arch. Another ligation (middle ligation) was made of mono-filament dissected from the 5 − 0 silk suture (soft and thin) then on the segment between these two ligations near to the proximal one. CCA was punctured using 33G insulin injection needle to create an entry on the arterial wall of the CCA, precisely between the proximal and middle ligations. Subsequently, the needle was withdrawn, and a 5 − 0 mono-filament (Prolene Ethicon) was cautiously introduced through the puncture site. The filament was advanced along the lumen of the artery, with particular attention to preventing penetration of the back artery wall. Once the tip of the filament reached the location of the distal ligation, the middle ligation was tighten to fix the filament and prevent bleeding from the puncture site. The distal ligation was loosened and removed totally to create an adequate vision for the following procedure. Subsequently, the filament was further advanced beyond the ICA-ECA bifurcation into the ICA. It was then progressed to perforate the Circle of Willis. Upon observing an increase in ICP, the filament was carefully withdrawn, repositioning the tip back to the middle ligation site before complete removal. The middle ligation was gently tightened once more, but not fully closed. At this juncture, controlled blood release ensued as the filament tip exited the puncture site. This controlled bleeding served the purpose of expelling air from the arterial lumen, thereby facilitating the formation of a blood clot at the puncture site. After short waiting period, typically less than one minute, the middle ligation could be removed, blood perfusion was reestablished via the contralateral circulation through the Willis circle. After approximately 2 to 3 min of waiting, the proximal ligation was gradually released, allowing observation of blood perfusion from the proximal to the distal portion of the artery and the detection of a clear arterial pulse. Once this state was confirmed, waiting for the blood clot to firmly formation at the puncture site for another 2 to 3 min, then the proximal ligation was fully removed. Throughout this procedure, the proximal ligation could be readily re-tightened in case of excessive bleeding, providing effective control over the bleeding process (Figs. 1, 4 and 5, Movie. S1).

The incision closure was made in a routine fashion, and the mice were kept in the cage over a heating pad and wet food on the floor of the cage to facilitate reaching. Surgical duration and ICP value were recorded for further investigation (Table 1; Figs. 1 and 2).

### Postoperative neurological assessment and brain samples autopsy

At postoperative day 1, the mice were evaluated by Rotarod test, open-field test, and body weight loss. Prior to conducting the neurological assessment, the body weight loss of the mice was measured and recorded with a precision of 0.1 g. The formula of body weight loss was (body weight preoperative - body weight postoperative) ÷ body weight preoperative × 100%.

The Rotarod test apparatus operated in an acceleration mode, featuring a rotational speed gradient from 4.0 to 40 RPM (Rotations Per Minute). Mice were then placed on the rotating rod and underwent testing until reaching either the point of falling from the rod to the base or the predetermined endpoint of 300 s, as outlined in our experimental protocol. Each mouse underwent this testing procedure thrice, and the ultimate outcome was derived from the average performance across these three trials^24^.

The open-field test were conducted within a cubic polyvinyl chloride box measuring 42 × 42 × 42 cm^25^. A 10-second video recording was performed using a stationary cell phone camera set at 30 frames per second (FPS). The recorded video clips underwent analysis using the open-source software “Tracker,” accessible at https://physlets.org/tracker/.

The Tracker software automatically processed data, encompassing the mouse’s movement trajectory and distances. Subsequently, this data was exported and stored for analysis. To facilitate the analysis, the distance data only employ the absolute value without units.

After the neurological assessment, all of the mice were euthanized by cardiac perfusion under deep anesthesia, the brain samples were harvested and evaluated (Fig. 2).

### Statistical analysis

Statistical analysis were conducted on ICP, surgical duration, success rate, mortality, Rotarod test, open-field test, and body weight loss within each group. All data are expressed as mean ± standard deviation except mortality and success rate. Fisher’s exact test was employed to compare success rates and mortality between groups, with statistical significance set at a *P* < 0.05. For comparing ICP, surgical duration, Rotarod test, open-field test, and body weight loss among different groups, one-way ANOVA was utilized to identify any statistically significant differences. A *P* of < 0.05 was considered indicative of statistical significance. All statistical analyses were executed using R Software (version 3.6).

## Electronic supplementary material

Below is the link to the electronic supplementary material.

Supplementary Material 1

Supplementary Material 2

## Data Availability

All data are available in the main text and the supplementary materials.

## Abbreviations

SAH: Subarachnoid Hemorrhage
ECA: External Carotid Artery
ICA: Internal Carotid Artery
CCA: Common Carotid Artery
ICP: Intracranial Pressure
cWp: Circle of Willis Perforation
OA: Occipital Artery
PPA: Pterygopalatine Artery
ACA: Anterior Cerebral Artery
MCA: Middle Cerebral Artery
MCAO: Middle Cerebral Artery Occlusion
RPM: Rotations Per Minute
FPS: Frames Per Second
