# Supplementary material for: A modified surgical approach to induce circle Willis perforation in mice using the common carotid artery
Source: Sci Rep. 2025 Apr 21;15:13769. doi: 10.1038/s41598-025-97603-1 (PMC12012005; doi:10.1038/s41598-025-97603-1)
Supplement: Supplementary file 2 — Supplementary Material 2 [file 41598_2025_97603_MOESM2_ESM.pdf]

Supplementary Materials of "A Modified Surgical Approach to Induce Circle Willis Perforation in Mice Using the Common Carotid Artery"

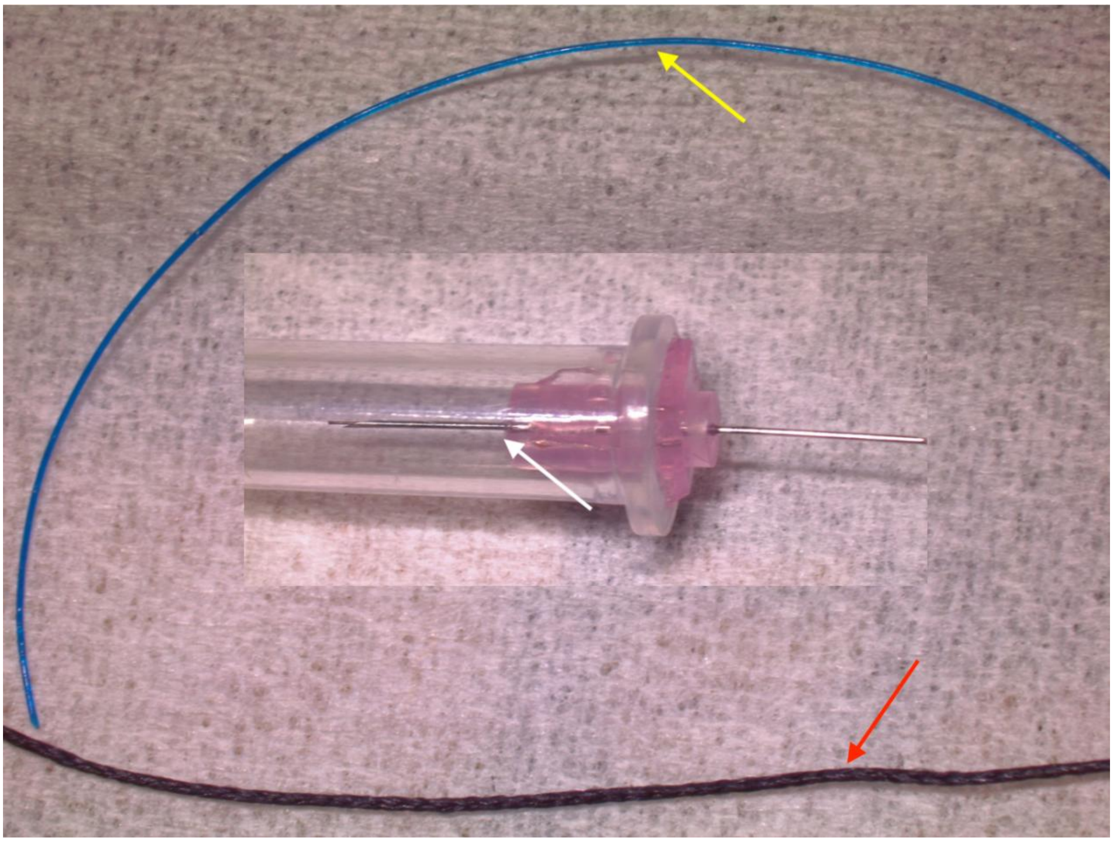

Fig. S1: Instruments Applied During the Common Carotid Artery Surgical Procedure

Fig. S1: The yellow arrow indicates the use of a 5-0 monofilament (Prolene, Ethicon, USA), the red arrow indicates a 5-0 silk suture (Ethicon, USA), and the white arrow marks a 33-gauge insulin injection needle (Nuofan, Ningbo, China).

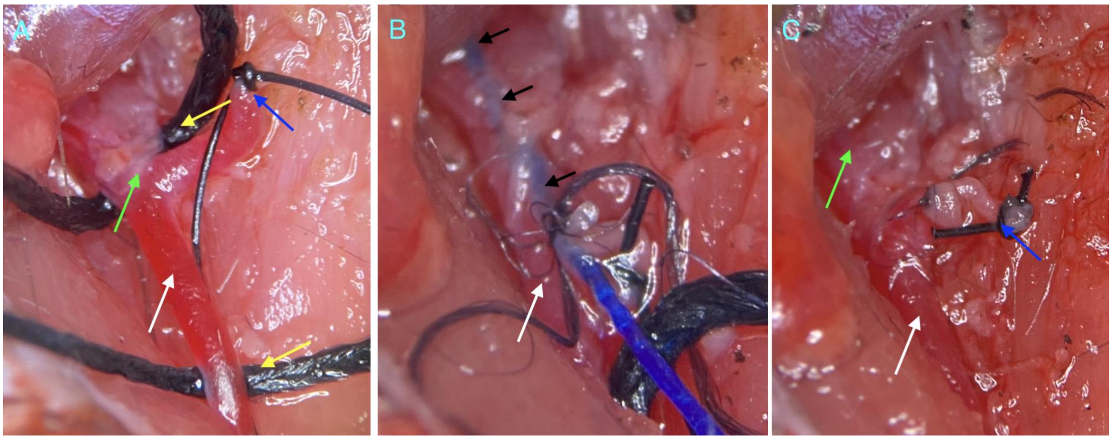

Fig. S2: Sequential Steps of the ECA Approach for the Circle Willis Perforation Subarachnoid Hemorrhage Model

Fig. S2A: The stump of the ECA is indicated by a blue arrow. Exposing the ECA-ICA bifurcation for facilitating filament guidance into the ICA (indicated by the green arrow). Two ligations (indicated by the yellow arrows) are located on the distal and proximal end of the CCA (indicated by the white arrow).

Fig. S2B: Insertion of the nylon filament (indicated by the black arrows) through the ECA stump, progressing into the ICA to complete the perforation. The white arrow indicates the CCA.

Fig. S2C: The ECA approach results in sacrificing the ECA indicated by the blue arrow. And the CCA (indicated by the white arrow) and ICA (indicated by the green arrow) resume the blood flow after the removal of the ligations.

ECA: External Carotid Artery; ICA: Internal Carotid Artery; CCA: Common Carotid Artery.

Movie S1： Surgical Procedure of The Common Carotid Artery Approach to Induce Subarachnoid Hemorrhage in Mice

Movie. S1 demonstrated the key steps of the surgical procedure, showing the whole process of inducing SAH in mice.

Video Link: https://youtu.be/yGFJJISvkg0
